# Cyberdiversity: Improving the Informatic Value of Diverse Tropical Arthropod Inventories

**DOI:** 10.1371/journal.pone.0115750

**Published:** 2014-12-26

**Authors:** Jeremy A. Miller, Joshua H. Miller, Dinh-Sac Pham, Kevin K. Beentjes

**Affiliations:** 1 Department of Terrestrial Zoology, Naturalis Biodiversity Center, RA Leiden, The Netherlands; 2 Department of Entomology, California Academy of Sciences, Golden Gate Park, San Francisco, California, United States of America; 3 Plazi, Zinggstrasse 16, Bern, Switzerland; 4 Department of Geology, University of Cincinnati, 500 Geology/Physics Building, Cincinnati, Ohio, United States of America; 5 University of Alaska Museum, Fairbanks, Alaska, United States of America; 6 Florida Museum of Natural History, University of Florida, Gainesville, Florida, United States of America; 7 Institute of Ecology and Biological Resources, Vietnam Academy of Science and Technology, Cau Giay Dist, Ha Noi, Vietnam; 8 Naturalis DNA Barcoding Facility, Naturalis Biodiversity Center, RA Leiden, The Netherlands; University of Colorado, United States of America

## Abstract

In an era of biodiversity crisis, arthropods have great potential to inform conservation assessment and test hypotheses about community assembly. This is because their relatively narrow geographic distributions and high diversity offer high-resolution data on landscape-scale patterns of biodiversity. However, a major impediment to the more widespread application of arthropod data to a range of scientific and policy questions is the poor state of modern arthropod taxonomy, especially in the tropics. Inventories of spiders and other megadiverse arthropods from tropical forests are dominated by undescribed species. Such studies typically organize their data using morphospecies codes, which make it difficult for data from independent inventories to be compared and combined. To combat this shortcoming, we offer cyberdiversity, an online community-based approach for reconciling results of independent inventory studies where current taxonomic knowledge is incomplete. Participating scientists can upload images and DNA barcode sequences to dedicated databases and submit occurrence data and links to a web site (www.digitalSpiders.org). Taxonomic determinations can be shared with a crowdsourcing comments feature, and researchers can discover specimens of interest available for loan and request aliquots of genomic DNA extract. To demonstrate the value of the cyberdiversity framework, we reconcile data from three rapid structured inventories of spiders conducted in Vietnam with an independent inventory (Doi Inthanon, Thailand) using online image libraries. Species richness and inventory completeness were assessed using non-parametric estimators. Community similarity was evaluated using a novel index based on the Jaccard replacing observed with estimated values to correct for unobserved species. We use a distance-decay framework to demonstrate a rudimentary model of landscape-scale changes in community composition that will become increasingly informative as additional inventories participate. With broader adoption of the cyberdiversity approach, networks of information-sharing taxonomists can more efficiently and effectively address taxonomic impediments while elucidating landscape scale patterns of biodiversity.

## Introduction

As biodiversity continues its unabated decline [1], [2], taxonomic and geographic biases constrain our ability to understand and predict the consequences of these losses and devise effective mitigation strategies [3], [4]. In terms of richness and abundance, arthropods dominate animal life, especially in the tropics. Yet vertebrates and vascular plants, both of which have comparatively low diversity, are the dominant study subjects for assessing the biological ramifications of anthropogenic perturbations and establishing conservation priorities [5]–[7]. While vertebrates have been shown to be poor surrogates for arthropod conservation priorities [5]–[7], the geographic distribution of arthropod species has been found to reliably predict the conservation priorities of vertebrates (i.e., optimizing networks of reserve areas to maximize the persistence of species [8]). This asymmetry arises largely because the geographic ranges and environmental tolerances of individual arthropod species tend to be more restricted than vertebrates or vascular plants, enabling megadiverse arthropod groups to track ecological gradients at finer spatial resolution. Because of their high richness and sensitivity to environmental variables, arthropods offer some of the finest-grained data with which to assess terrestrial biodiversity at individual localities (alpha [α] diversity) and changes across landscapes (beta [β] diversity) [5], [9]. However, one of the greatest impediments to the broader use of arthropod communities for studying and maintaining global biodiversity is our current profoundly incomplete and geographically biased data on fundamental taxonomy. Substantial proportions of arthropods, especially those found in tropical regions, have yet to be formally described and lack scientific names [10]. Yet, names are the mechanism by which data about a species (including geographic distribution) are aggregated. The process of naming and describing species, however, is time-consuming. While online innovations are increasing the pace of species descriptions and improving accessibility to taxonomic data, these advances come at a time when investment and training in taxonomy is declining [11], [12]. If the fine-grained pattern of arthropod biodiversity is to be broadly integrated into conservation assessment, it is apparent that we need to diminish dependence on formal scientific names. To accommodate this need, an alternative model is emerging designed to share biodiversity data that is not yet ready for formal taxonomic publication so researchers may efficiently and effectively evaluate and integrate that information with other data [13], [14].

Two technological advances are revolutionizing standard practice in inventories of arthropods (including spiders): (1) the increased accessibility of digital photomicroscopy, and (2) DNA barcoding. Perhaps the most precocious online collection of digital images of a diverse arthropod taxon is AntWeb (http://www.antweb.org/). At the time of this writing, this global effort offers photos of nearly 16,000 ant species freely available online along with specimen occurrence data. Unidentified ant species referred to only by morphospecies codes (rather than valid Linnaean binomials) in some published studies are also included [15]. AntWeb gives independent researchers working in the same region an opportunity to determine which species – including species not yet formally described – are shared between independent studies without the need to physically examine vouchers. Photographs and specimen occurrence records can provide much of the determination power we expect from formal taxonomic literature, even when these resolve to morphospecies codes rather than Linnaean names. DNA barcoding offers an independent method of species identification and classification. This approach involves building a library of sequences from a standard region of the genome to aid species identification and discovery [16]. For animals, the ∼648 base pair region of the mitochondrial gene cytochrome oxidase I has become the dominant barcode marker [16], [17]. DNA barcoding as an enterprise has strengths and limitations, and these have been the subject of spirited debate [18]–[33]. Within this debate, some have argued convincingly that data from multiple independent sources (e.g., morphology, DNA sequences) should be considered (e.g., [19], [20], [22], [23], [26], [27], [31]–[33]). Given that classification based on either data source alone fails some of the time, disagreement between approaches indicates the need for focused study to resolve the conflict [19], [20], [34]. By extension, determining the number of species within and shared between ecological inventories based on a combination of morphological and molecular sequence data is preferable to relying on either method alone. Species-level taxa determined on the basis of combined morphological and DNA sequence data (i.e., without formal names) are referred to as integrated operational taxonomic units (IOTUs) [35].

**Figure 1:**
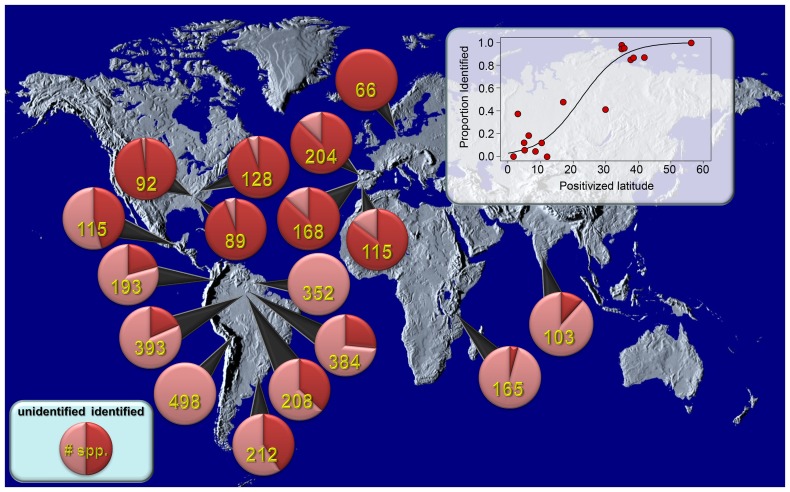
Structured inventories of spiders from around the world. Red portion of each pie chart represents species identified according to formal nomenclature in the original publication; the remaining pink portion represents those identified to morphospecies. Total observed species richness is in yellow. Inset shows significant relationship between distance from the equator (expressed as positivized latitude) and the proportion of identified species (logistic regression; β = 0.113 (β_se_ = 0.0488, e^β^ = 2.306), *p* = 0.0211, McFadden's R^2^ = 0.72). Studies with a high proportion of identified species (which are largely found in temperate regions) are relatively easy to evaluate for community similarity, while studies in regions with more poorly developed taxonomy (e.g., the tropics) may not be as easily reconciled. This roadblock can be bypassed using the cyberdiversity framework, which allows data on the whole community to be made publically available, and can foster reconciliation of independent inventories, including those with high proportions of undescribed species. Data from Colombia [62], Brazil [63]–[65], Denmark [54], Guyana [39], India [66], Mexico [67], Peru [56], Portugal [57]–[59], Tanzania [68], and United States [55], [60], [61]. In some cases, specimens representing morphospecies from these studies have been subsequently described in taxonomic publications [112], [113].

Spiders are one of the richest orders of life on earth, and structured inventories (i.e., surveys that use replicable sampling protocols with multiple complimentary collecting methods) are fundamental sources of data about species richness [36]–[38]. However, inventories in temperate regions have significantly higher proportions of their species identified with scientific names than inventories in tropical regions (Fig. 1). This is perhaps not surprising given the relatively low number of species and high intensity of taxonomic research at temperate latitudes. The consequence of this taxonomic imbalance is that data from temperate inventories may be far more readily integrated with existing knowledge compared to tropical inventories. In regions with relatively undeveloped taxonomic literature, ecological studies typically categorize unidentified species using "morphospecies" concepts [39]–[41]. This means using the skills of a morphological taxonomist to classify individuals in the collection without depending on incomplete and fragmentary taxonomic literature. This approach is sufficient for elucidating biodiversity patterns within a particular study, but makes it cumbersome to compare results between independent studies. Conscientious investigators typically deposit voucher specimens in museum or university collections, which means that morphospecies from different studies can be reconciled, but doing so is often prohibitively time consuming. As a consequence, independent biodiversity studies on the same taxa in a single region have limited capacity to build on each other or to document biological patterns beyond the scope of each individual study.

### The cyberdiversity approach

Cyberdiversity is an online approach to facilitate species recognition regardless of taxonomic determination status. Web-based tools may include collections of digital images, DNA sequences, or ideally both. The cyberdiversity approach engages several recognized impediments to the understanding of fundamental aspects of megadiversity and the wider adoption of arthropod data for conservation assessment. These challenges include (a) the large number of species that remain undescribed, (b) the scientific ignorance concerning the geographic distribution, abundance, and environmental sensitivities of most species, and (c) the lack of awareness of invertebrate conservation issues in the social-political spheres [10]. Photographs and DNA barcodes, organized by persistent unique identifier, allow data on the geographic distribution and abundance of species to be compiled whether or not the species have scientific names [13], [14]. The ready availability of these data through online databases makes the effort visible to a range of stakeholders. Following a central principal of biodiversity informatics, the primary data are formatted according to community standards and digitally exposed in ways that allow them to be aggregated, recombined, and repurposed [42]–[44].

**Figure 2:**
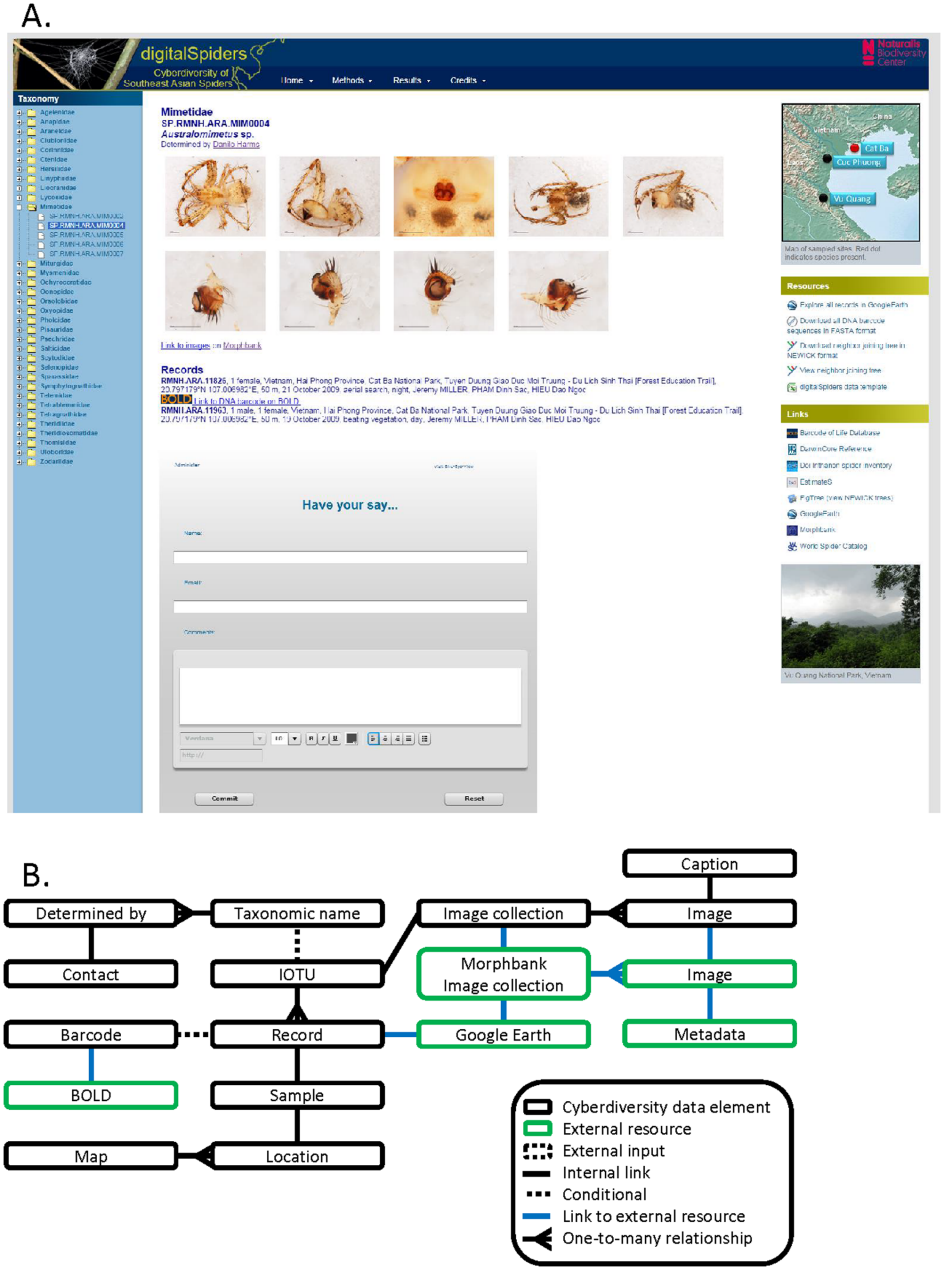
Data visualization and schematic data relationships on the digitalSpiders cyberdiversity web site. (A) The digitalSpiders website is designed around a three-panel layout. The left panel is a taxonomic index to IOTUs. Once an IOTU is selected, the center panel shows the taxonomic name (where available), a collection of images, collection records, and a utility for submitting and viewing public comments. Individual images are linked to higher resolution versions on Morphbank annotated with extensive metadata. The image collection for the IOTU is also linked to a corresponding image collection on Morphbank. Records may be associated with a DNA barcode sequence and linked to the BOLD database. All records can be viewed through Google Earth with markers linked to collections of images on Morphbank. New content can be submitted using the digitalSpiders data template. (B) Data relationships are configured around an IOTU identified by a unique species code. Each IOTU is associated with one or more records. Each of those records is associated with a sample taken at a location. The map is derived from the set of unique locations. All records can be viewed through Google Earth with markers linked to collections of images on Morphbank. Records may be associated with a DNA barcode sequence and linked to the BOLD database. Each IOTU is associated with a collection of one or more images, linked to a corresponding image or image collection on Morphbank. Where an IOTU is associated with a valid taxonomic name, this determination is credited to one or more taxonomists along with their contact information.

Here, we introduce a new, expandable cyberdiversity resource: www.digitalSpiders.org, currently populated with data from our three 2009 inventories of Vietnamese spiders (Fig. 2). The web site layout features a three-panel design consisting of (1) a taxonomic navigation tree organized by IOTU within family, (2) species identifier (unique IOTU code, and where available taxonomic name with determination credit), images, records, links to DNA barcode sequences (where available), and user comments, and (3) a map indicating species presence for each inventory location. The three panel design permits simultaneous viewing of morphology and occurrence data, delivering with a single click the most critical information needed to reconcile future inventory data with that currently available on the web site. Unfortunately, the leading collaborative biodiversity data environment, Scratchpads (http://scratchpads.eu/), does not currently support such a layout. The digitalSpiders site also makes all records available through Google Earth with markers linked to collections of images on Morphbank. In the Google Earth environment, records can be filtered to display any combination of records or IOTUs. New content can be submitted using the digitalSpiders data template, subject to validation by the site administrator.

Ours is the first structured tropical spider inventory study to include DNA barcode data for most of the sampled community. Fortunately, it is not the first study from the region to make libraries of morphospecies images available online. Images of all morphospecies resulting from a 2003 survey of spiders from Doi Inthanon National Park, Chang Mai Province, Thailand have also been posted online (http://aracnologia.macn.gov.ar/ThaiPlot/). This inventory was conducted in October, the same time of year as our Vietnamese study, by an independent research team. The results of the Doi Inthanon study have not been published in the form of a scientific journal article. Nevertheless, because the leaders of this study chose to post images of their morphospecies online, we were able to rapidly assess characteristics of change in spider communities (β-diversity) across ca. 1,000 km.

The cyberdiversity approach is a call to researchers to share data that facilitate the reconciliation of inventory results across studies, multiplying the number of sites available for analysis. The ultimate goal is to accumulate enough reconciled inventory points to meaningfully model patterns of diversity on regional, continental, and even global scales. With data from only four sites currently available for comparison (three in Vietnam, one in Thailand), we cannot yet provide a definitive analysis of large-scale biodiversity patterns. Instead, we present a series of preliminary analyses to demonstrate the kinds of questions and diversity parameters that can be addressed once more inventories are reconciled.

### Modeling horizon: landscape-scale change in megadiverse communities

Once we have assessed community similarity across sites, we can begin to focus on quantifying β-diversity and identifying its geographic and climatic drivers. We present a preliminary demonstration of this in a distance-decay framework [45], [46], where community change (i.e., pair-wise community similarity) is modeled as exponential decay functions of both the geographic and climatic (WorldClim, http://www.worldclim.org/bioclim [47]) distances between sites. Using this exponential decay framework, we can then estimate the species turnover rate; specifically, the geographic or climatic distance across which half the species are different from one point to another (halving distance; d_0.5_, which is constant across space). We can also estimate the similarity of communities at small distances, specifically the initial similarity (*s*_0_), when distance between community samples equals zero. With a sufficient number of sites, variance partitioning can be used to disentangle covariance between geographic and climatic change [48], thought we do not attempt this here. Our preliminary analyses are intended to demonstrate the kinds of questions that are possible to explore with reconciled inventory data.

## Results

**Figure 3:**
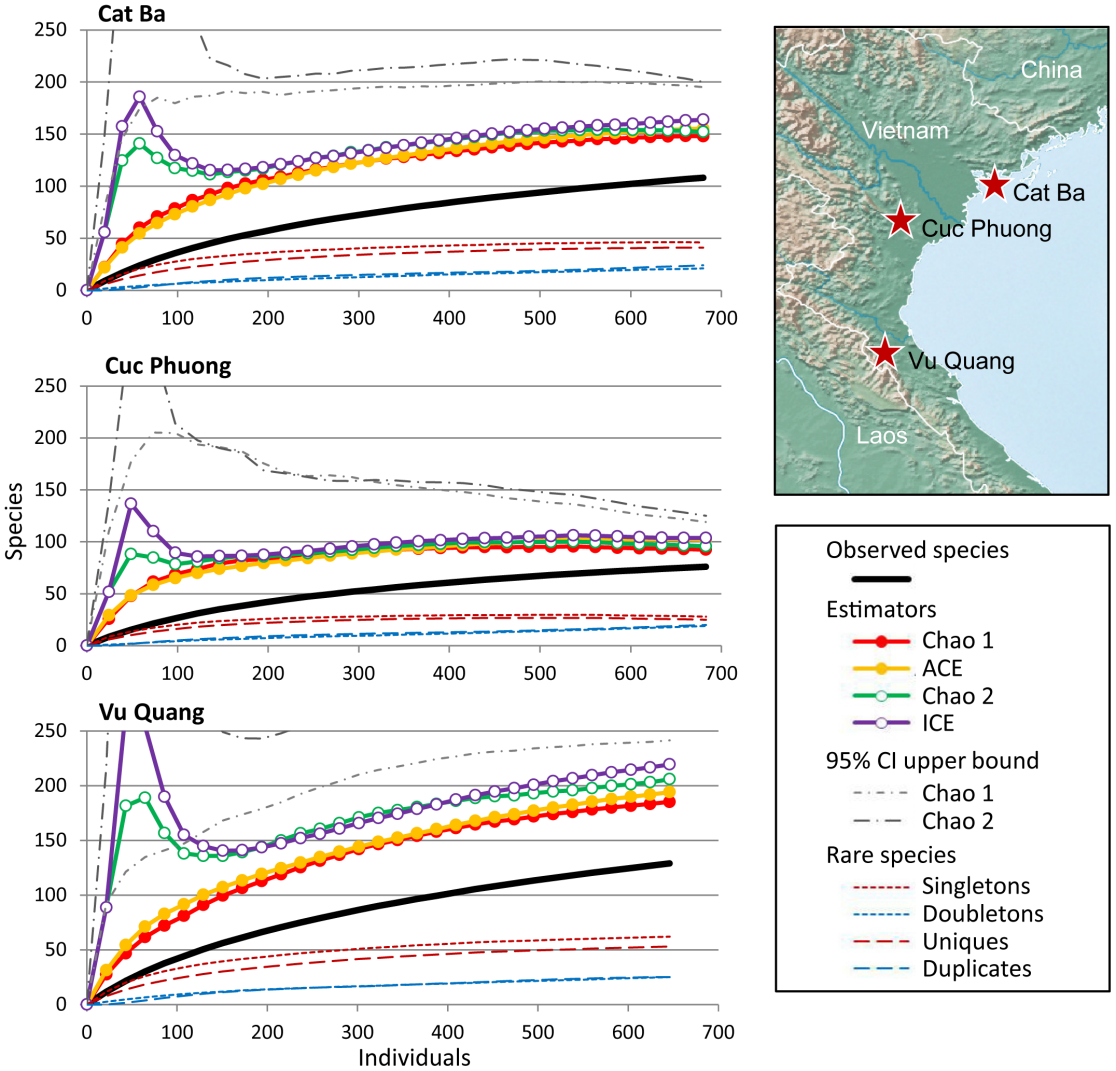
Species richness estimation curves with map showing locations of Vietnamese study sites. Data include all adult spider specimens collected from one hectare plots in three Vietnamese National Parks: Cat Ba, Cuc Phuong, and Vu Quang. The number of species observed from each inventory, and the number of singletons, unique, doubletons, and duplicates in each inventory are given, as well as four non-parametric estimators of sample completeness: Chao 1, ACE, Chao 2, and ICE (upper 95% confidence intervals provided for Chao 1 and Chao 2). Estimated Inventory completeness is variable, but none are complete. Hence, assessment of community similarity based on these inventories should account for unobserved shared species.

Our surveys of three study sites in Vietnam yielded 2,009 adult spiders comprising 240 species. Non-parametric species richness estimators (Chao 1, Chao 2, ACE, and ICE) indicate that the Cuc Phuong inventory was the closest to completion (range of mean estimated completeness [hereafter, estimated] 73–82%). The Cuc Phuong inventory yielded the lowest number of observed species (76) and the highest number of individuals (683), so the sampling intensity (the number of individuals per species) was comparatively high (9.0). The Cat Ba inventory was only slightly less complete (estimated 66–73%). The observed species richness in Cat Ba was considerably higher than Cuc Phuong (108) for almost the same number of individuals (680), so the sampling intensity was correspondingly lower (6.3). The Vu Quang inventory was the richest of the three sites with 128 observed species from a sample of only 646 individuals (sampling intensity 5.1). Non-parametric estimators suggest that this inventory was 60–72% complete (Table 1, Fig. 3).

**Table 1:** Observed and estimated species richness for the three rapid inventories of spiders in Vietnam.

		Estimated richness [estimated completeness of observed sample]			
Site	Observed Richness	Chao 1 (upper 95% C.I.)	ACE	Chao 2 (upper 95% C.I.)	ICE
Cat Ba	108	148 (195), [73% (55%)]	155, [70%]	152 (200), [71% (54%)]	164, [66%]
Cuc Phuong	76	93 (119), [82% (64%)]	102, [74%]	96 (125), [80% (61%)]	104, [73%]
Vu Quang	128	178 (229), [72% (56%)]	188, [68%]	197 (262), [65% (49%)]	213, [60%]

All three inventories recovered largely different faunas with pairs of sites sharing only 29–31 observed species; 17 species were observed at all three sites. The proportion of observed shared species (the Jaccard index) across site pairs ranges from 0.14–0.20. Chao's estimated proportional similarity (see Methods) ranges from 0.13–0.19 with fairly wide 95% confidence intervals (Table 2).

**Table 2:** Observed and estimated similarity of sampled spider communities in Southeast Asia.

Site pair	Jaccard similarity (shared/combined species)	Estimated shared species (95% C.I.)	Estimated combined species	Chao's Estimated Proportional Similarity (95% C.I.)
Cat Ba - Cuc Phuong	0.20 (31/153)	45 (31, 65)	212	0.21 (0.14, 0.34)
Cat Ba - Vu Quang	0.14 (29/207)	42 (29, 73)	302	0.14 (0.10, 0.27)
Cat Ba - Doi Inthanon	0 (0/214)	0 (0, 0)	287	0 (0, 0)
Cuc Phuong - Vu Quang	0.17 (29/175)	44 (29, 93)	247	0.18 (0.11, 0.47)
Cuc Phuong - Doi Inthanon	0.0056 (1/181)	1 (1, 19)	233	0.005 (0.004, 0.090)
Vu Quang - Doi Inthanon	0.031 (7/227)	12 (7, 32)	309	0.037 (0.022, 0.109)

Estimated combined species is the sum of ACE for both sites minus the estimated shared species.

DNA barcode sequences were obtained from 176 of the 240 species in the study (73%). Sequencing was attempted on 531 specimens (26% of the collection), 372 of which (70%) yielded a barcode sequence. In total, DNA sequences were obtained for 19% of the collected specimens. The BIN (Barcode Index Number) algorithm [49], which partitions barcode sequences into species-like taxonomic units (independent of morphology), suggests the barcodes obtained for this study represent 188 species, a net increase of 12 species compared to the results based on the combination of morphological and molecular sequence data (i.e., IOTUs).

**Figure 4:**
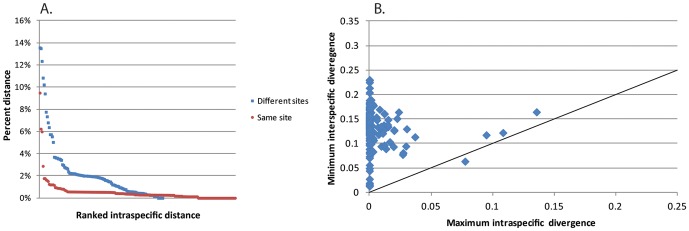
Discriminatory power of DNA barcodes. (A) Within-species genetic distances (within-site [red], between-site [blue]) ranked by magnitude. (B) The barcode gap expressed as the maximum within-species distance compared to the minimum between-species distance; line shows equal interspecific-intraspecific distances. The magnitude of intraspecific genetic distance is variable across species, but maximum intraspecific distance is almost always less than the minimum interspecific genetic distances. Distance modeled using the Felsenstein 1984 model [50], which, using the IOTU classification, optimizes these data according to the Akaike Information Criterion. See supplementary documents for the same data modeled using Kimura 2-parameter and uncorrected *p* distances.

Intraspecific variation in the barcode sequence was assessed based on 73 species for which more than one individual was successfully sequenced. Of these, within-site variability was assessed using 192 conspecific pairwise comparisons from 63 species, and between-site variability was assessed using 121 conspecific pairwise comparisons from 33 species. Overall, conspecific distances between sites (based on the optimal Felsenstein 1984 [50] model) were considerably higher than within sites (Mann-Whitney test, U = 3901, z = −9.908, *p* = 0.0001), suggesting geographic population structure (Figs. 4A and S1 in S1 File).

**Figure 5:**
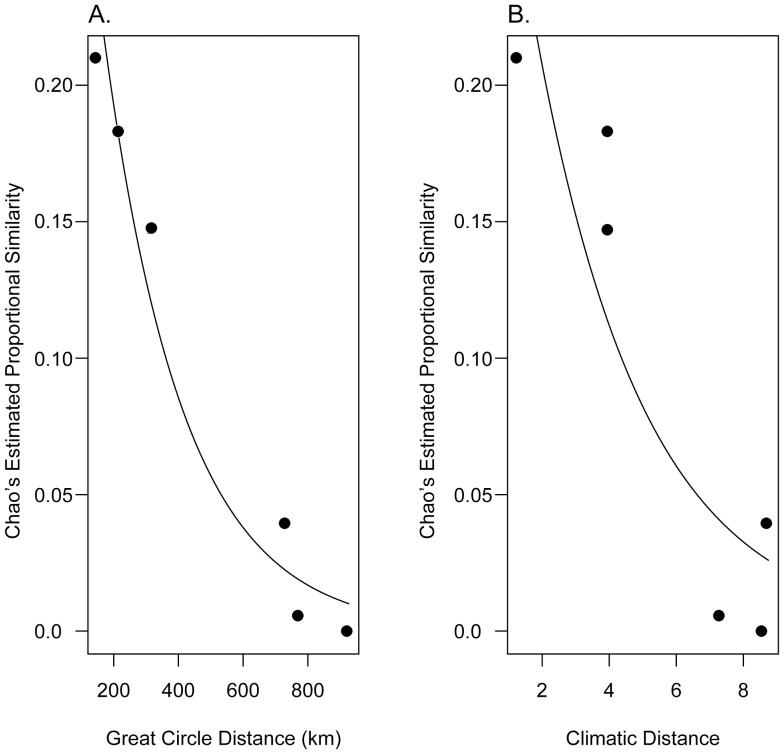
Distance decay of community similarity. Pair-wise community similarity for Southeast Asian spider communities and their decay against (A) geographic and (B) climatic distances. Similarity is significantly correlated with both geographic and climatic distances (mantel tests; *p*<0.05). For geographic distance, the estimated community halving distance is 171 km. With additional inventory sites, variance partitioning could be used to disentangle covariance between geographic and climatic change.

We find that community similarity (Chao's estimated proportional similarity) for Southeast Asian spiders is significantly correlated with geographic distance (Mantel test r = 0.99, *p* = 0.042), and that the community halving distance based on all available data is 171 km (Jackknifed mean (mean_J_) and standard error (SE_J_): 192±111 km) (Fig. 5). Initial similarity (i.e., similarity at 0 distance, *s*_0_) was 0.43 (mean_J_ ± SE_J_: 0.48±0.17). Across the study area, the 19 climatic variables and altitude data are highly correlated, with the first two principal component axes characterizing 99% of the variance (Table S1 in S1 File). Based on a combined distance matrix of those first two principal component axes, we find that Chao's estimated proportional similarity is also significantly correlated with change in climate among sites (Mantel test r = 0.95 *p* = 0.044). Using all available data, initial similarity based on climate was close to that calculated for geographic distance: *s*_0_ = 0.38, but the jackknifed mean and standard error was higher (mean_J_ ± SE_J_: 0.68±0.48). The halving distance for climate is d_0.5_ = 2.25 (mean_J_ ± SE_J_  = 3.00±3.77), which corresponds to a 93.2% (range across ± SE_J_: 80.2–100%) decay in community similarity across the maximum distance of the sampled region. This is within error of the estimated change in community similarity based on the halving distance for geographic change: 96.5% (88–100%).

Cobra analysis (see Methods, Table 3) revealed both strengths and inefficiencies in our sampling regime. The largest number of sampling hours at all sites were devoted to AEN (searching for spiders in the aerial stratum at night), and this proved to be the most efficient method for species discovery in all cases. In Cat Ba (12 hours), AEN was relatively saturated; in Vu Quang (9 hours), more samples could have been allocated to this method; Cuc Phuong (10 hours) was intermediate. WIN (extraction of arthropods from sifted leaf litter using Winkler traps) ranked second in field hours at all sites (two hours spent sifting leaf litter per sample), and was the second most efficient method in two sites (Cat Ba and Cuc Phuong). WIN at Cat Ba (10 hours) and Vu Quang (8 hours) approached relative saturation, but more WIN samples could have been allocated at Cuc Phuong (8 hours). BED (beating vegetation during the day) ranked third or fourth in efficiency and fourth (or tied for fourth) in effort. BED included moderately to highly efficient samples and more effort could have been usefully allocated here, especially at Cat Ba. LDD (searching for spiders on the ground during the day; 4–6 samples per site) contributed enough unique species to the inventory that it includes some high scores in the Cobra analysis, but most of the species contributed to the inventory using this method would have been collected with fewer samples. LDN (searching for spiders on the ground at night; 6–8 samples per site) was the least efficient method, and included several sample hours at each site that contributed little to the inventory; too much effort was devoted here.

**Table 3:** Allocation of samples by method for the three rapid inventories of spiders in Vietnam with totals of adult specimens and species by method and site.

		Method					
Site		BED	AEN	LDD	LDN	WIN	Total
Cat Ba	Samples	6	12	4	8	5	35
	Adults	121	196	50	55	258	680
	Species	29	34	12	17	18	108
Cuc Phuong	Samples	4	10	4	6	4	28
	Adults	44	258	133	92	156	683
	Species	11	28	16	17	14	76
Vu Quang	Samples	5	9	6	6	4	30
	Adults	64	265	169	56	92	646
	Species	19	36	21	18	15	128

BED: beating vegetation during the day; AEN: searching for spiders in the aerial stratum at night; LDD: searching for spiders on the ground during the day; LDN: searching for spiders on the ground at night; WIN: extraction of arthropods from sifted leaf litter using Winkler traps.

## Discussion

### Cyberdiversity takes on the Taxonomic Impediment

Today's urgent need for better biodiversity knowledge is mismatched by the relatively slow pace of taxonomic progress for tropical spiders and other megadiverse groups. Using online databases to increase the recognizability of species regardless of whether they have a scientific name is one way to mitigate this asymmetry [13], [14]. For example, on the digitalSpiders web site, each IOTU page features a comments field to facilitate discussion and contribute to taxonomic identification (Fig. 2). Cyberdiversity resources can also serve to stimulate traditional taxonomy; taxonomic specialists can browse the online collection of images and data, find specimens relevant to their research, and request specimen loans and/or aliquots of extracted genomic DNA. With the participation of a broad network of contributors and taxonomists, the cyberdiversity approach can even improve the description rate of the undescribed portion of our global fauna [51]. In an era of biodiversity crisis, climate change, and other challenges, the scientific and public spheres have common interest in synergies that make research products more responsive to the questions of the day. Thus, practices that make it easier to compare and combine data across different inventory studies are highly desirable for deriving the maximum information value from our research investment. We encourage authors who include specimens and DNA extracts featured on cyberdiversity platforms in their research to follow an open access cybertaxonomic publication model [52], [53]. All data shown on digitalSpiders are protected by a creative commons license, meaning they can be used for third party research provided the original source is cited and derivative works are distributed according to a similar license. A healthy ethic of data sharing can advance research across the community. We ask authors to think carefully about the benefits they derive from shared data and acknowledge accordingly those responsible if they hope to foster this incipient trend.

### Sampling strategy and assessment

During structured inventory surveys, a selection of semi-quantitative field sampling methods is typically applied to estimate spider species richness [36], [39], [54]–[67]. Each sampling method in a structured inventory targets a different portion of the fauna, which may overlap to a greater or lesser extent with other methods. Given that inventories have limited resources, decisions about how to partition effort among the sampling methods can have a major impact on the ultimate completeness of the inventory. To assess and improve sampling design, Cardoso [38] developed a method for optimizing allocation of sampling effort for spider inventories based on data from three studies conducted in Portugal. His method is based on a *post hoc* randomization analysis of inventory data to determine the optimum allocation of sampling effort by method to maximize species encountered.

**Figure 6:**
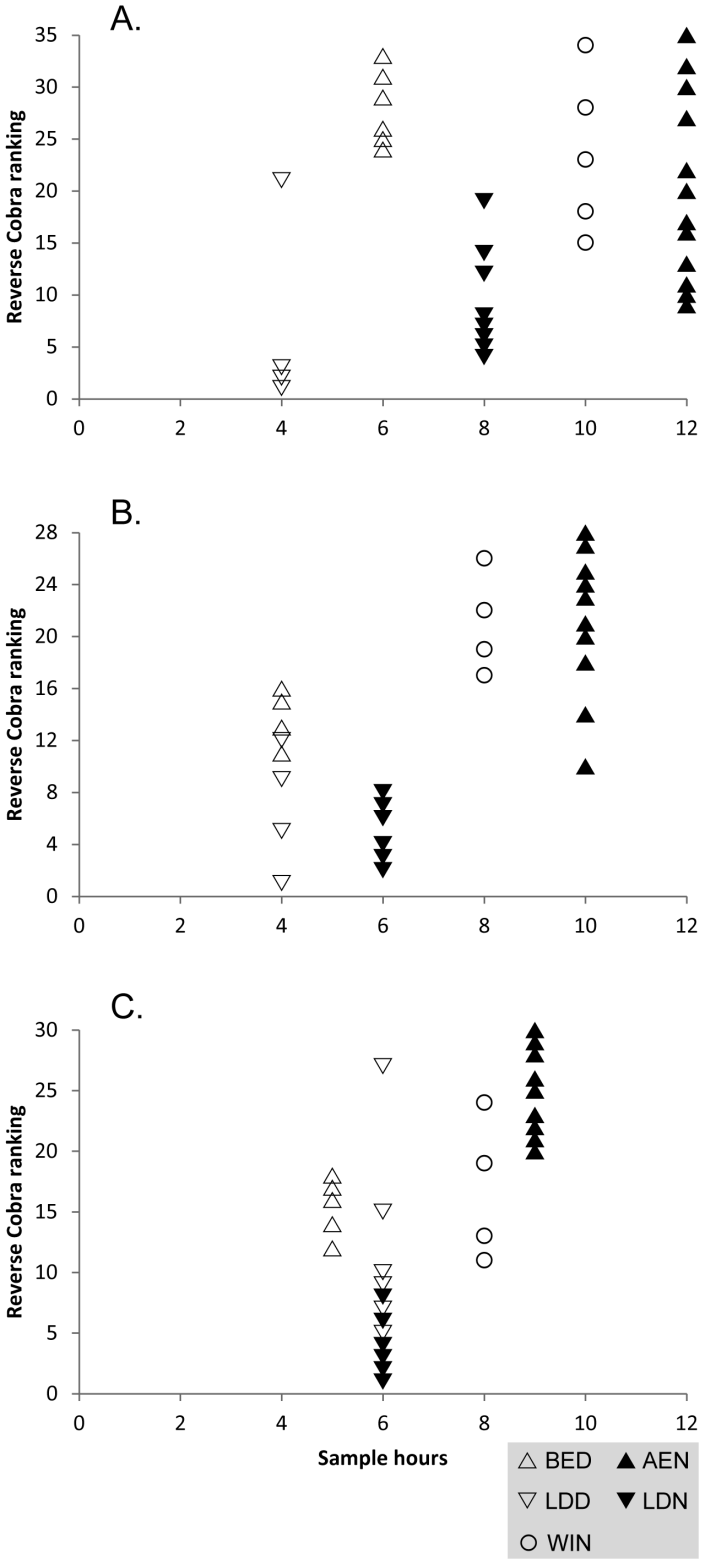
Efficiency of sampling methods compared to hours of field time allocated. Sampling efficiency was scored using the reverse Cobra ranking (see text) for (A) Cat Ba, (B) Cuc Phuong, and (C) Vu Quang. Methods: BED: beating vegetation during the day; AEN: searching for spiders in the aerial stratum at night; LDD: searching for spiders on the ground during the day; LDN: searching for spiders on the ground at night; WIN: extraction of arthropods from sifted leaf litter using Winkler traps. Methods conducted during daylight hours are unfilled, methods conducted during night hours are black-filled. Methods targeting above-ground strata are up-pointing triangles, methods targeting ground strata are down-pointing triangles or a circle (WIN). Each WIN sample required two field hours; samples using all other samples were one hour each. High reversed Cobra scores indicate maximum efficiency of a method for contributing species to the inventory; low scores indicate sampling saturation for that method.

Based on results from previous tropical forest inventory studies [39], [68], our structured inventory sampling strategy allocated more time to AEN than any other single collecting method. Cobra analysis [38] confirmed that AEN was the most efficient method for species discovery (Fig. 6). WIN, which is used in standard inventories of some groups other than spiders [69], was also found to be an efficient method for spiders. These results are in contrast to those found for Portuguese spiders [38], where pitfall traps and sweeping were the most efficient methods for species accrual and AEN was less crucial. However, direct comparison between Cardoso [38] and our inventories is complicated due to mismatches in sampling methods: WIN was not included in the Portuguese inventories and neither sweeping nor pitfalls were included in our Vietnamese inventories. Sweeping would seem *a priori* to be a dubious investment in the generally thorny and herb-poor Vietnamese forests, and pitfalls were omitted because of our regrettable failure to locally obtain propylene glycol preservative, which facilitates DNA extraction from pitfall specimens [70]. Note that the protocol presented by Cardoso [38] is not intended as a global standard and it is acknowledged that efficient sampling in other regions, including tropical forests, probably requires a different sampling strategy.

### DNA barcodes for species discrimination

Within the BOLD framework, DNA barcode sequences are assigned to species based on cluster analysis and empirically derived interspecific distance thresholds (BIN [49]). The distance threshold for animals is typically set at 2–3% sequence divergence [71], [72]. Barrett and Hebert [73] reported that a 2% divergence threshold was adequate for discriminating spider species. We found that 2–3% divergence was indeed sufficient to assign most barcodes to species. However, conspecific distances were considerably higher for a few species, especially species shared between sites (Figs. 4A and S1A, C in S1 File). There are several possible explanations for this, including (a) taxonomic error (some IOTUs in this study may actually represent more than one species), (b) inadvertent amplification of a nuclear pseudogene of mitochondrial origin [18], and (c) bacterial infection (e.g., *Wolbachia*), which can distort patterns of mitochondrial variation and inheritance [74]. Alternatively, high conspecific genetic distances may simply reflect variable intra-species divergence, with most species characterized by small (e.g., <2%) divergences and a few species characterized by higher divergences (Figs. 4A and S1A, C in S1 File). Species sorting based on integrated analysis of DNA and morphology (IOTUs) found fewer species than the algorithmic approach [49] based on sequence data alone. Based on IOTU designations and barcode sequences, minimum genetic distances between species were almost always larger than distances within species (Figs. 4B and S1B, D in S1 File). The incongruence between DNA-only (BIN) and the integrated approach (IOTU) is almost certainly attributable to the many rare species that characterize our inventories; without a reasonable estimate of within-species variation, genetic gaps between species can be obscure [75]. The system of IOTUs presented here is subject to testing and refinement by future studies. Thus, an additional advantage of the cyberdiversity approach is the ability to readily integrate new data with legacy data to test and refine the findings of previous studies. Both IOTU designations and DNA-only BIN codes [49] are included in the supporting information to highlight incongruence between the two approaches and facilitate future re-assessment (S1 Appendix).

### Modeling community change

The proportion of shared species is a useful and intuitive concept for comparing two communities. When inventories are nearly complete, the Jaccard index expresses this adequately. But when sampling is incomplete, especially in communities of megadiverse taxa with large proportions of rare species, the Jaccard can underestimate the proportion of shared species [76]–[78]. Shared rare species present the most significant challenge because they are most likely to be missed in one or both inventories. To account for unobserved shared species [76], [79], we replace the observed values in the Jaccard with estimated ones (see methods). We use the ACE [80] to estimate richness of each community because the shared species estimator is an extension of the ACE [77], [78]. We call this Chao's estimated proportional similarity. Note that combining estimated values in this way can inflate the variance of the resulting community similarity estimate [79], [81]. Chao's estimated proportional similarity should not be confused with Chao's abundance-based Jaccard index [76], [79], which does not report an estimated proportion of shared species (unless abundances of all species are equal). One reason why we concentrate on presence-absence (as opposed to including relative abundance data) is that inventories of spiders and other diverse arthropods typically employ an assortment of field sampling methods, each targeted to a subset of the fauna. Thus, the relative abundances of species sampled in this way are not expected to be representative of their actual abundances within communities [82]; random sampling of individuals from ecologically and morphologically diverse species communities in structurally complex habitats is usually not realistic. While species rarity is useful in the context of non-parametric species richness and shared species estimators, diversity measures that rely on community species abundance distributions (e.g., Chao's abundance-based Jaccard index) may be problematic in this context.

Chao's estimated proportional similarity is significantly correlated with both geographic and climatic distances (mantel tests; *p*<0.05). Nevertheless, it is clear that our understanding of regional and comparative β-diversity will greatly profit from an expanded dataset. In addition, future analyses involving more reconciled inventories should use variance partitioning to disentangle contributions of distance and climate to β-diversity pattern [48]. While acknowledging the preliminary nature of this analysis, to our knowledge this is the first quantification of the geographic rate of species turnover for tropical spider communities. Future analyses can also test for differences in the root causes of biodiversity structuring across landscapes. For example, data from angiosperms suggest stronger relationships between community similarity and geographic distance (as opposed to climate differences), suggesting a biodiversity pattern largely shaped by dispersal ability and climate history, particularly the Pleistocene glaciation of North America and Europe [48], [83], [84]. We are curious if spiders and other megadiverse arthropod groups with comparatively narrow environmental tolerances and fast generation times follow a similar pattern, or if historical relicts such as glaciations are more quickly obliterated in such communities.

Similarly, there is an enduring interest in distinguishing the root causes of the spatial structuring of biodiversity (β-diversity). Concordance between community composition and environmental conditions suggest biodiversity structuring driven by niche-sorting, while concordance with geographic distance invokes dispersal abilities, landscape characteristics, and neutrality [45], [46], [85]–[90]. Whether the β-diversity patterns of spiders and other megadiverse arthropods accord or contrast with other communities of organisms, increased availability and analysis of data based on diverse taxa with fine-grained climatic and spatial structuring will contribute to a richer understanding of these fundamental biological questions. In addition, a more complete understanding of the spatial structure of megadiverse communities, including their geographic, climatic, and latitudinal components, can improve the targeting of conservation priorities and provide quantitative estimates of biodiversity structuring across the globe [91]. The cyberdiversity approach will help us to realize the full complement of scientific and conservation benefits offered by structured inventories of megadiverse taxa in a rapid and rigorous manner.

## Methods

### Ethics statement

All necessary permits were obtained for the described study, which complied with all relevant regulations. A specimen collecting permit was granted by the Vietnam Administration of Forestry. All samples were collected in national parks. No protected species were involved in this study. An export permit to allow sample processing was granted by the Institute of Ecology and Biological Resources, Vietnam Academy of Science and Technology. Specimens have been divided between the Institute of Ecology and Biological Resources in Hanoi and the Naturalis Biodiversity Center in Leiden in accordance with an agreement made prior to the expedition (S1 Appendix).

### Sampling and processing

Spiders were sampled from one-hectare plots in forest habitat [36], [39], [68] established in three Vietnamese national parks: Vu Quang, Cuc Phuong, and Cat Ba. Five sampling methods were used: (1) beating vegetation during the day (BED), (2) searching for spiders in the aerial stratum at night (AEN), (3) searching for spiders on the ground during the day (LDD), (4) searching for spiders on the ground at night (LDN), and (5) extraction of arthropods from sifted leaf litter using Winkler traps (WIN; www.entowinkler.at). Searching and beating methods were conducted in one-hour blocks; leaf litter sifting was done in two hour blocks plus a minimum drying time of 48 hours. Allocation of samples by method for each of the three inventories is reported in Table 3.

After field sampling, adult spiders were roughly sorted to morphospecies. When there was any question as to whether particular specimens belonged to one or more morphospecies, they were initially treated as different. These cases were later re-examined in light of DNA sequence data (it is easier to merge data from multiple putative morphospecies into one than to partition a hodgepodge). Morphological and barcode data were reconciled to create a collection of integrated operational taxonomic units (IOTUs) [35]. One or more specimens of every IOTU was photographed, both sexes when available. Photographs were made using a Nikon DS-Ri1 camera mounted on a Leica M165 C stereoscope operated using NIS Elements software. Images from multiple focus planes were combined and edited in Syncroscopy Auto-Montage software version 5.03 (http://www.syncroscopy.com). Images (1877 from 532 specimens representing all 240 species) and associated collection data were uploaded to Morphbank (www.morphbank.net; S2 Appendix).

### DNA Barcoding

Tissues from 1–4 legs were sent to the Naturalis DNA barcoding facility. Specimens for DNA barcoding were selected to represent both sexes of all species from all sites, as available. All DNA voucher specimens were photographed. Extractions were performed using either the Qiagen DNEasy Blood and Tissue kit or the Macherey-Nagel NucleoMag Tissue kit (http://www.mn-net.com/) on the Thermo Labsystems KingFisher extraction robot.

Following initial tests using a variety of primer combinations, PCR was performed using the primers LCO1490 (5′-GGTCAACAAATCATAAAGATATTGG-3′) [92] and Chelicerate Reverse 2 (5′-GGATGGCCAAAAAATCAAAATAAATG-3′) [73]. PCR reactions contained 18.75 µl mQ, 2.5 µ 10× PCR buffer CL, 1.0 µl 25 mM of each primer, 0.5 µl 2.5 mM dNTPs and 0.25 µl 5 U Qiagen Taq. PCR was performed using initial denaturation of 180 s at 94°C, followed by 40 cycles of 15 s at 94°C, 30 s at 50°C and 40 s at 72°C, finished with a final extension of 300 s at 72°C and pause at 12°C. Sequencing was performed by Macrogen (http://www.macrogen.com). For all barcoded specimens, sequences and collection data were uploaded to the Barcode of Life Database (BOLD; http://www.boldsystems.org/; S3 Appendix).

DNA barcode sequences were aligned using ClustalW [93] with default parameters as implemented in DAMBE [94], [95]. Neighbor joining distances were calculated in DAMBE with 10,000 replicates of random terminal input order. Three different models were applied: Kimura 2-parameter [96], because of its widespread use in the DNA barcoding literature [71], uncorrected *p*-distances [97], and the optimal model as determined using the Akaike information criterion to evaluate models implemented in jModelTest (version 0.1.1) [98].

For all three models, the barcode gap was expressed as the minimum interspecific distance against the maximum intraspecific distance [97] and also the pair-wise intraspecific sequence distances within sites and between sites.

### Sampling strategy assessment

We used the sampling optimization method described by Cardoso [38], [99] to assess our sampling design efficiency. Inventory data from each of the three Vietnamese sites was analyzed using Cobra with 1000 randomizations. This program estimates the order of samples by method that will produce the greatest number of species with the least sampling effort. To compare the optimized sampling strategy to the actual allocation of field hours, we reversed the order of samples (so efficient samples received high values) and plotted this reverse Cobra ranking against the number of actual field hours devoted to each method at each site. High points on the reverse Cobra ranking score indicate the sampling of maximum efficiency for contributing new species to an inventory; low points indicate sampling saturation (i.e., inefficiency for discovering new species). Efficient, unsaturated methods warrant more resources during field sampling. Note that each Winkler sample was based on two hours of daylight field time spent sifting leaf litter, so each Winkler sample was counted as two hours.

### Biodiversity analysis

Species richness for each site was estimated according to two abundance-based (Chao 1 and ACE) and two incidence-based (Chao 2 and ICE) non-parametric estimators [80], [100]–[103] as implemented in EstimateS [104]. In all cases, the classic (not the bias corrected) formula was used following the *post hoc* recommendations given by EstimateS. Ninety-five percent confidence intervals for Chao 1 and Chao 2 were also calculated [100]. Community similarity between sites was calculated as: 
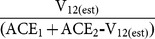
where V_12(est)_ is Chao's estimated shared species between community 1 and community 2 (for communities where species discovery probabilities are heterogeneous; substituting Chao 1_-shared_ or Chao 1_-shared-bias corrected_ as appropriate) [77], [105], and ACE_1_ and ACE_2_ are the abundance-based coverage estimates of species richness in community 1 and community 2 [80] as implemented in SPADE [106]. This estimated proportional similarity is modeled after the Jaccard index:



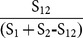
where S_1_ and S_2_ are the observed species richnesses in communities 1 and 2, and S_12_ is the observed number of shared species between communities. Thus, Chao's estimated proportional similarity is equivalent in composition to the Jaccard except it replaces empirical counts with non-parametric estimates of those values. Upper and lower bounds of the 95% confidence interval for V_12(est)_ was used to estimate variability of the similarity measure.

We modeled β-diversity as an exponential decay of community similarity (species turnover) across (great circle or climatic) difference [45], [46], [48], [83], [107]–[109] using the distance-decay model:
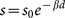
where *s* is the community similarity (estimated here using Chao's estimated proportional similarity), *d* is the distance between sites, and *s*_0_ (initial similarity, when *d* = 0) and β (decay constant) are modeled parameters. While it is common for distance-decay studies to estimate *s*_0_ and β using a linear regression of log(*s*) on distance, our dataset includes pairs of sites that have no species in common (*s* = 0), which results in an undefined portion of the regression (log(0) is not defined). While it is possible to add a small value to these zero-points during the calculation (or remove such comparisons from the model) [45], [46], [48], [83], these manipulations result in meaningful changes to resulting estimates of *s*_0_ and β [107]. Because community similarities range between 0 and 1, to include *s* = 0 site pairs, we, instead, modeled distance-decay as binomial proportions using a generalized linear model with a log link function [107].

By parameterizing our exponential diversity-decay model, we gain two pieces of biological insight: (1) an estimate of the similarity of two samples taken from, essentially, the same locality (*s*_0_; when *d* = 0), which provides some indication of sample completeness, underlying diversity, and habitat heterogeneity, and (2) the distance across which community similarity decays by half, (the “halving distance”, d_0.5_):
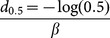



Because of the nature of exponential curves, this halving distance is independent of placement along the curve. Thus, we gain an estimate of a fundamental characteristic of species turnover along any stretch of space or climate across the sampled region.

Because our calculations are conducted on paired-comparisons, traditional estimates of model fit and standard errors of parameter estimates are not valid. Thus, we use a jackknife approach [107] to calculate the standard error of parameter estimates. Our jackknife successively removes each of our *n* sites (not simply site-pairs) and re-runs our analyses *n* - 1 times. The variance of the parameters is then calculated as the total sum-of-squares divided by *n* jackknifed values multiplied by (*n* - 1)/*n* [107], [110]. Finally, we use mantel tests (using 10,000 permutations) to calculate the significance of relationships between community similarity and distance (geographic or climatic).

Climate data were obtained from the 30 arc second rasters of 19 bioclimatic variables and a digital elevation model from WorldClim (http://www.worldclim.org/current, generic global grids, version 1.4 [47]). While we are fundamentally interested in testing how changes in climate and elevation influence patterns of β-diversity, our current sample size is too small to exhaustively explore their impacts. Additionally, the available 19 BioClim climatic variables are highly autocorrelated (86% of the 190 pair-wise climate and elevation comparisons from Southeast Asian sites have a Pearson correlation greater than 0.5, 60% have correlations greater than or equal to 0.8). To summarize the available climate data into fewer variables, we performed a principal component analysis (PCA; Table S1 in S1 File). Climate data were log-transformed, mean-centered, and scaled prior to analysis. To calculate climatic distances among sites, we then calculated a Euclidean distance matrix using the scores from the first *j* principal component axes that cumulatively summarize more than 95% of the variance. Because geographic and climatic distance are expressed in different units, direct comparisons of d_0.5_ are difficult, although comparisons of *s*_0_ between climate and geographic datasets are not affected. To compare community change, we calculate the estimated community change between the two furthest points. That is, we calculate how many d_0.5_'s will have occurred across the sampled landscape and estimate that proportional change in overall spider community as:
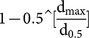
where d_max_ is the maximum distance (geographic or climatic) observed by any paired site comparison. Community distance decay and associated analyses were scripted in R [111].

## Supporting Information

S1 File **This file contains Table S1 and Figure S1.** Figure S1, Discriminatory power of DNA barcodes under alternative models. A, C, within-species distances ranked by magnitude and partitioned into distances between individuals sampled from the same site (red) and distances between individuals sampled from different sites (blue). B, D, the barcode gap expressed as the maximum within-species distance against the minimum between-species distance. Distance models (based on IOTU classification): A, B, Kimura 2-parameter; C, D, uncorrected *p*. Table S1, Results of PCA analysis of environmental data (WorldClim) derived from the three Vietnamese and one Thai inventory sites. Variable loadings on the first two principal components (which cumulatively explain 99% of the variance) are also provided.
(DOC)

S1 Appendix **Primary specimen occurrence data.** Includes catalog numbers, IOTU codes (taxonID), DNA barcode identification numbers (BOLD BIN), specimen location (institutionID), and complete specimen-by-sample data for this study. Fields follow Darwin Core standards (http://rs.tdwg.org/dwc/) where applicable.
(XLSX)

S2 Appendix **Images available on Morphbank (** www.morphbank.net **).**
(XLSX)

S3 Appendix **DNA barcode sequences available on BOLD (** www.boldsystems.org/ **).**
(XLSX)
