# Supplementary material for: Cyberdiversity: Improving the Informatic Value of Diverse Tropical Arthropod Inventories
Source: PLoS One. 2014 Dec 26;9(12):e115750. doi: 10.1371/journal.pone.0115750 (PMC4277369; doi:10.1371/journal.pone.0115750)
Supplement: S1 File — This file contains Table S1 and Figure S1. Figure S1, Discriminatory power of DNA barcodes under alternative models. A, C, within-species distances ranked by magnitude and partitioned into distances between individuals sampled from the same site (red) and distances between individuals sampled from different sites (blue). B, D, the barcode gap expressed as the maximum within-species distance against the minimum between-species distance. Distance models (based on IOTU classification): A, B, Kimura 2-parameter; C, D, uncorrected p. Table S1, Results of PCA analysis of environmental data (WorldClim) derived from the three Vietnamese and one Thai inventory sites. Variable loadings on the first two principal components (which cumulatively explain 99% of the variance) are also provided. (DOC) [file pone.0115750.s001.doc]

**Supporting Information: Cyberdiversity: improving the informatic value of diverse tropical arthropod inventories**

**Jeremy A. Miller ^1,2,3,*^, Joshua H. Miller ^4,5,6^ , Dinh-Sac Pham ^7^, Kevin Beentjes ^8^**

**1** Department of Terrestrial Zoology, Naturalis Biodiversity Center, Postbus 9517, 2300 RA Leiden, The Netherlands, **2** Department of Entomology, California Academy of Sciences, 55 Music Concourse Drive, Golden Gate Park, San Francisco, California 94118, USA, **3** Plazi, Zinggstrasse 16, Bern, Switzerland, **4** Department of Geology, University of Cincinnati, 500 Geology/Physics Building, Cincinnati, Ohio 45221, United States of America, **5** University of Alaska Museum, Fairbanks, Alaska 99775, United States of America, **6** Florida Museum of Natural History, University of Florida, Gainesville, Florida 32611, United States of America, **7** Institute of Ecology and Biological Resources, Vietnam Academy of Science and Technology, Hoang Quoc Viet Road. Cau Giay Dist, Ha Noi, Vietnam, **8** Naturalis DNA Barcoding Facility, Naturalis Biodiversity Center, Postbus 9517, 2300 RA Leiden, The Netherlands

^*^ E-mail: jeremy.miller@naturalis.nl

**Figure S1. Discriminatory power of DNA barcodes under alternative models.**

**Table S1.** **Results of PCA analysis of environmental data (WorldClim) derived from the three Vietnamese and one Thai inventory sites.**


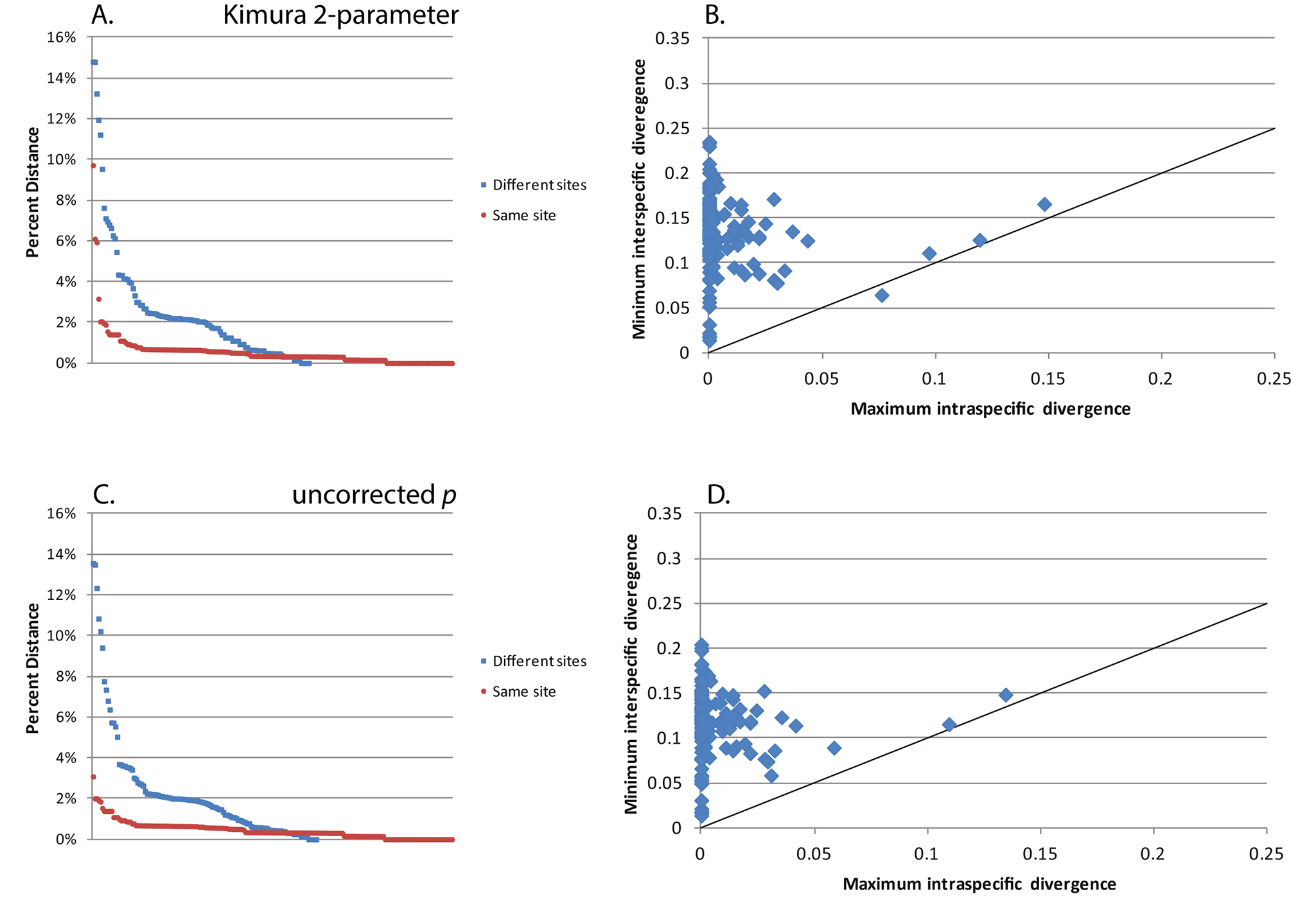


**Figure S1. Discriminatory power of DNA barcodes under alternative models.** A, C, within-species distances ranked by magnitude and partitioned into distances between individuals sampled from the same site (red) and distances between individuals sampled from different sites (blue). B, D, the barcode gap expressed as the maximum within-species distance against the minimum between-species distance. Distance models (based on IOTU classification): A, B, Kimura 2-parameter; C, D, uncorrected *p*.

**Table S1.** **Results of PCA analysis of environmental data (WorldClim) derived from the three Vietnamese and one Thai inventory sites.** Variable loadings on the first two principal components (which cumulatively explain 99% of the variance) are also provided.

|  |  | **Loadings**  **(Cumulative proportional variance explained)** | |
| --- | --- | --- | --- |
| **Variable code** | **Environmental variable description** | **PC1**  **(0.82)** | **PC2**  **(0.99)** |
| BIO1 | Annual Mean Temperature | 0.995 | -0.0666 |
| BIO2 | Mean Diurnal Range (Mean of monthly (max temp - min temp)) | -0.945 | -0.307 |
| BIO3 | Isothermality (BIO2/BIO7) (× 100) | -0.878 | -0.479 |
| BIO4 | Temperature Seasonality (standard deviation × 100) | 0.95 | 0.294 |
| BIO5 | Max Temperature of Warmest Month | 0.99 | -0.0812 |
| BIO6 | Min Temperature of Coldest Month | 0.998 | -0.0101 |
| BIO7 | Temperature Annual Range (BIO5-BIO6) | -0.977 | 0.0592 |
| BIO8 | Mean Temperature of Wettest Quarter | 0.99 | 0.116 |
| BIO9 | Mean Temperature of Driest Quarter | 0.975 | -0.194 |
| BIO10 | Mean Temperature of Warmest Quarter | 0.996 | 0.011 |
| BIO11 | Mean Temperature of Coldest Quarter | 0.97 | -0.241 |
| BIO12 | Annual Precipitation | 0.992 | -0.0169 |
| BIO13 | Precipitation of Wettest Month | 0.993 | -0.0816 |
| BIO14 | Precipitation of Driest Month | 0.537 | -0.839 |
| BIO15 | Precipitation Seasonality (Coefficient of Variation) | 0.626 | 0.76 |
| BIO16 | Precipitation of Wettest Quarter | 0.947 | 0.321 |
| BIO17 | Precipitation of Driest Quarter | 0.641 | -0.768 |
| BIO18 | Precipitation of Warmest Quarter | 0.876 | 0.482 |
| BIO19 | Precipitation of Coldest Quarter | 0.622 | -0.783 |
| ALT | Elevation | -0.973 | 0.158 |
